# Multiparameter flow cytometric detection and quantification of senescent cells *in vitro*

**DOI:** 10.1007/s10522-020-09893-9

**Published:** 2020-08-10

**Authors:** Adeolu Badi Adewoye, Dimitris Tampakis, Antonia Follenzi, Alexandra Stolzing

**Affiliations:** 1 Centre for Biological Engineering, School of Mechanical, Electrical and Manufacturing Engineering, Loughborough University, Loughborough, LE11 3TU UK; 2 Institute of Inflammation and Ageing, College of Medical and Dental Sciences, University of Birmingham, Birmingham, B15 2TT UK; 3 Division of Cancer Studies, King’s College London, London, UK; 4 Department of Health Sciences, Università del Piemonte Orientale “A. Avogadro,”, 28100 Novara, Italy

**Keywords:** Senescence, Mesenchymal stem cells, Aging, Flow cytometry, Quantification

## Abstract

It has been over half a century since cellular senescence was first noted and characterized, and yet no consensus senescent marker has been reliably established. This challenge is compounded by the complexity and heterogenic phenotypes of senescent cells. This necessitates the use of multiple biomarkers to confidently characterise senescent cells. Despite cytochemical staining of senescence associated-beta-galactosidase being a single marker approach, as well as being time and labour-intensive, it remains the most popular detection method. We have developed an alternative flow cytometry-based method that simultaneously quantifies multiple senescence markers at a single-cell resolution. In this study, we applied this assay to the quantification of both replicative and induced senescent primary cells. Using this assay, we were able to quantify the activity level of SA β-galactosidase, the expression level of p16^INK4a^ and γH2AX in these cell populations. Our results show this flow cytometric approach to be sensitive, robust, and consistent in discriminating senescent cells in different cell senescence models. A strong positive correlation between these commonly- used senescence markers was demonstrated. The method described in this paper can easily be scaled up to accommodate high-throughput screening of senescent cells in applications such as therapeutic cell preparation, and in therapy-induced senescence following cancer treatment.

**Electronic supplementary material:**

The online version of this article (doi:10.1007/s10522-020-09893-9) contains supplementary material, which is available to authorized users.

## Introduction

Emerging evidence suggests that the accumulation of senescent cells in tissues and organs may underlie ageing and age-related diseases (Krishnamurthy et al. 2004; Chen et al. 2007; Yanai and Fraifeld 2018). For instance, accumulation of senescent cells has been reported in ageing primate, rodent, and human tissues (Dimri et al. 1995; Herbig et al. 2006; van Deursen 2014). This has also been observed in age-related diseases such as type 2 diabetes (T2DM), musculoskeletal disorders, and neurodegenerative and cardiovascular diseases (Muñoz-Espín and Serrano 2014; Olivieri et al. 2018). Several studies have suggested a correlation between the proliferation potential of mesenchymal stem cells and the age of donors (Choudhery et al. 2014; Stolzing et al. 2008; Virant-Klun et al. 2019). Furthermore, cells derived from Werner syndrome patients exhibit premature senescence in comparison to cells from age-matched healthy individuals (Cheung et al. 2014). Rapidly accumulating evidence from these studies and others has enhanced our understanding of the role of senescent cells in the ageing process.

Of course, cellular senescence is no doubt an essential cellular process that modulates various aspects of life such as tumour suppression, wounding healing, and embryonic development (Chuprin et al. 2013; Coppé et al. 2008; Demaria et al. 2014). However, the detrimental effects of senescent cells have also been reported in age-related diseases. Recent findings show that senescent cells can negatively impact their immediate microenvironment through the senescence-associated secretory phenotype (SASP) (Coppé et al. 2010; Ovadya and Krizhanovsky 2014). It is also known that through these secreted proteins, senescent cells may have a stimulatory effect on tumour cell proliferation and migration (Coppé et al. 2010). Several lines of evidence suggest clear health benefits of reducing the accumulation of senescent cells. Such benefits include delayed onset of age-related diseases, the extension of healthspan (length of time lived in good health), and enhanced cardiac stress tolerance (Xu et al. 2015; Roos et al. 2016; Baker et al. 2016). This observation suggests senescent cells could be a promising therapeutic target for age-related degenerative diseases, and in enhancing healthy ageing. Thus, developing sensitive and precise detection methodology as well as being able to quantify senescent cells in an easy-to-perform assay would enhance research and translational studies.

The complexity and heterogeneity of senescence make it challenging to develop a universal biomarker for precise identification and quantification of senescent cells. Currently, the senescence-associated beta-galactosidase (SA-β-Gal) staining at pH 6.0 (Dimri et al. 1995) remains the widely used method to measure senescence. Increased SA-β-Gal activity is reported in replicative and stress-induced senescence in various cell types (Debacq-Chainiaux et al. 2009). Despite its wide applications, the assay is plagued with limitations such as confluency (Severino et al. 2000), serum starvation (Yegorov et al. 1998), time-consuming, and operator bias. These limitations can potentially increase the likelihood of false-positive results in senescent cell screening. Given the phenotypic heterogeneity of senescent cells, no single marker can reliably identify or quantify senescence. In this study, we have developed a robust flow cytometry-based multiparameter senescence assay that accurately detects and quantifies senescent cells in culture. Our approach combined three biomarkers with two label-free additional markers, that allow rapid, quantitative, and high-throughput detection of senescent cells. We tested this method on induced senescence generated by exposing adult mesenchymal stem cells, endothelial cells, and a cancer cell line to doxorubicin and 5’-Aza- 2 deoxycytidines and on long term cultured cells - replicative senescent cells.

## Materials and methods

### Reagents

Doxorubicin (Doxo) purchased from Cell Signaling Technology UK (5927); DDAOG (9H-(1,3-Dichloro-9,9-Dimethylacridin-2-One-7-yl) β-d-Galactopyranoside) from ThermoFisher Scientific UK, D6488; Bafilomycin A1 from Fisher Scientific UK, 10295441 and 5’-Aza- 2 deoxycytidines (Aza) from Sigma-Aldrich UK, A3656. All the chemicals were reconstituted in dimethyl sulfoxide (DMSO) as a stock solution. Antibodies used were as follows: PerCP-Cy™5.5 Mouse Anti-H2AX (pS139) from BD Biosciences, 564,718; Anti-CDKN2A/p16^INK4a^ [EPR1473] (Phycoerythrin) from Abcam ab209579, and Fixable viability Dye 450 from BD Biosciences UK, 562,247. All flow cytometry buffers BD Perm/Wash (554,723); BD Cytofix/Cytoperm (554,722) were purchased from BD Biosciences.

### Cell culture

Three different cell types were used: (a) mesenchymal stem cells (MSCs), derived from human dental pulp (DP-MSCs); (b) Blood Outgrowth Endothelial Cells (BOECs) supplied by Dr. Joris Braspenning at University Hospital of Würzburg and Prof. Antonia Follenzi at Università del Piemonte Orientale; (c) human glioblastoma cell line – U373 MG purchased from European Collection of Authenticated Cell Cultures (ECACC). MSCs were grown in Dulbecco’s modified Eagle medium (Sigma-Aldrich UK UK) supplemented with 10% of Fetal Bovine Serum (FBS) in standard condition (37 °C with 5% CO_2_). BOECs were grown under standard conditions in medium provided by Dr. Joris Braspenning at the University Hospital of Würzburg. U373 was cultured in modified Eagle’s medium-α (MEM-α) (Gibco 12,561,056; Thermo Fisher Scientific, Waltham, MA, USA) supplemented with 10% Fetal Bovine Serum (FBS; Gibco 12,483,040; Thermo Fisher Scientific, Waltham, MA, USA).

### Senescence induction

Senescence was induced by exposure of proliferating cells to either 200 nM Doxorubicin (Doxo) or 5 µM of 5’-Aza- 2 deoxycytidines (5-Aza) dissolved in DMSO for 48 h. Control samples were treated with equal molar DMSO in parallel with the treated cells. The cells were washed with PBS following incubation with the drugs or DMSO and cultured with fresh drug -free media for an additional 5 days before determining senescence level. All experiments were replicated and performed with appropriate controls.

### Cytochemical detection of SA-β-Gal

The SA-β-Gal activity was detected using a cytochemical approach according to (Dimri et al. 1995). Briefly, cells were fixed with 4% paraformaldehyde in PBS for 15 min at room temperature. The cells were washed twice with PBS and incubated overnight in fresh SA-β-gal staining solution containing 1 mg/ml 5-bromo-4-chloro-3-indolyl-b-Dgalactopyranoside, 5 mM potassium ferrocyanide, 5 mM potassium ferricyanide, 150 mM NaCl, 2 mM MgCl_2_, and 0.1 m phosphate buffer, pH 6.0 in darkness at 37 °C without CO_2_. For positive staining controls, fixed cells were treated with the same solution, but at pH 4.0, alongside with the experimental samples. Staining and imaging were done on a Leica optical microscope.

### Flow cytometric detection of SA-β-Gal using DDAOG substrate

In the presence of βGal, DDAOG substrate is hydrolysed to DDAO, which generates a far-red fluorescent signal. The staining protocol was adopted from Gong and colleagues with slight modifications (Gong et al. 2009). Briefly, cells were seeded into culture flasks (BD FALCON) cultured in DMEM containing 10% FBS for 24 h and subsequently treated with 0.1 µM Bafilomycin A1 to induce lysosomal alkalinisation and 0.02 mM DDAOG for 2 h at 37 °C. Cells were washed twice in PBS, detached by TryplE (Gibco, Life Technologies, USA) at 37 °C for 5 min for harvesting. The cells were stained for viability with a fixable viability dye (FV450; 1:1000) in PBS for 15 min at room temperature. They were then washed with 1× BD Wash buffer, followed by a 5-min centrifugation at 300×*g* before surface marker staining. Cells were then permeabilised and fixed with 200 µl BD Cytofix/Cytoperm solution for 30 min at room temperature. After incubation, the cells were washed twice in 1× BD Perm/Wash buffer before intracellular staining with antibodies. A 5 µl/sample of PerCP-Cy™5.5 Mouse Anti-H2AX (pS139 BD Biosciences) and 1:500 Anti-CDKN2A/p16^INK4a^ (EPR1473, Abcam, ab209579) antibody was added and incubated at room temperature for 30 min in the dark. Cells were washed twice in 1× BD Perm/Wash buffer prior to analysis on BD FACSCanto II flow cytometer (Beckton Dickinson, BD Bioscience).

### Data acquisition and analysis

Flow cytometry experiments were performed on BD FACSCanto II at the Centre for Biological Engineering, Loughborough University; data was analysed in FlowJo V10. After appropriate spectral compensation was performed, a minimum of 10,000 events per replicate was collected for analysis. Three replicates for each condition were analysed in each independent experiment and included an appropriate Fluorescence Minus One control (FMO). The data was expressed as the percentage of the total cell population that was positive for a given biomarker.

### RNA isolation and cDNA synthesis

Total RNA was extracted from senescent and proliferating cells using Direct-zol RNA Kits with in-column *DNAse* I treatment (Zymo Research, R2072) according to the manufacturer’s protocol. RNA concentration was determined on a NanoDrop 2000 spectrophotometer (Thermo Scientific) at an absorbance ratio of 260/280 nm, with an acceptable range of 1.9 to 2.2 applied. The quality was assessed on Agilent 2100 Bioanalyzer, and only samples with RIN of 9 or above were used for cDNA synthesis. cDNA was generated from 1 µg of the isolated RNA using a SensiFast cDNA synthesis kit (Bioline, BIO-65,053) according to the manufacturer’s instructions.

### Quantitative real-time PCR

The expression of senescence-associated genes (p16, p53, Ki-67, and p21) was analysed. Primer sequences used for the qPCR experiment are listed in Table 1 of the supplementary data. All qPCR experiments were performed on Step one Plus (Applied Biosystems) with SYBR Green detection chemistry. Each reaction contains 5 µl of 10-fold diluted cDNA, 7.5 µl of 2x SensiFast SYBR mix, 0.5 µM of each primer, and 1 µl nuclease-free water in a final reaction volume of 15 µl. The cycling parameters were: 1 cycle of 95 °C for 15 min, 40 cycles of 95 °C for 10 s and 60 °C for 30 s, followed by a melt curve. The amplification of the expected PCR product was confirmed with a single peak melting curve for each primer set. A standard curve of cDNA serial dilutions was generated to determine amplification efficiency for each primer set. All reactions were performed in four replicates and normalised to *36B4* expression. The 2^−△△Ct^ method (Wong and Medrano 2005) was used to calculate the relative expression level of each gene against the housekeeping gene *36B4.*

### Immunophenotyping and BOECs tube formation

Cells were tested for the expression of specific surface markers by flow cytometry, using the MSCs phenotyping kit (Miltenyi Biotec). This kit consists of two antibody cocktails: phenotyping (CD73-APC, CD90-FITC, CD105-PE, and CD34/CD45/CD14/CD20-PerCP) as an isotype control cocktail. Briefly, MSCs (5 × 10^5^ cells) were suspended in staining buffer consisting of 2 mM EDTA and 3% FBS in PBS and incubated for 10 min at 4 °C with either phenotype or isotype antibody cocktails. Data was acquired with the BD FACSCanto™ II (BD Biosciences) flow cytometer and analysed using Flowjo software version 10 (Flowjo). A compensation matrix was generated using single antibodies from the kit. Doublets were excluded using FSA vs. FSH gating for all samples before gating on total live cells, negative for 450 viability dye (BD Biosciences). For BOECs, anti-human monoclonal antibodies used included: anti-CD45-PE, anti-CD34-PE (ImmunoTools), anti-CD31-APC (ImmunoTools), anti-Tie2-PE and anti-VE-Cadherin-PE (all other antibodies from Miltenyi Biotec). For BOECs angiogenesis, cells were cultured on Cultrex PathClear Basement Membrane Extract (Biotechne) at 37 °C for 16 h. Evidence of tube-forming ability exhibited by the cells was captured via microscope imaging.

### Multiplex ELISA assay

The secretion of IL8, IL6, and MCP1 proteins in the culture medium was measured using Luminex technology according to the manufacturer’s instructions (R&D Systems). The culture medium was collected for each condition prior to senescence detection. Samples were centrifuged at 300×*g* for 5 min, and the supernatant was stored at − 80 °C until analysis. Samples were diluted 1:3 in PBS buffer and used at a volume of 100 µl/well in triplicates for each condition. Data acquisition was performed on a Luminex Magpix instrument; data was normalised to cell number for each condition.

### Statistical analysis

Each experiment was repeated at least three times unless otherwise stated in the figure legends. *p* < 0.05 was considered as statistically significant, as indicated with asterisks in the figures. All statistical analysis and graph plotting were conducted in GraphPad Prism 8 (GraphPad Software, La Jolla, CA, USA).

## Results

We began by characterising the cells used in this study and examining their senescence phenotypes according to the three senescence models applied. Analysis of replicative senescence (model 1) was performed on long-term cultured cells at passages 3–5 (early), 10–13 (intermediate) and 22–30 (late) in DP-MSCs. For induced senescence, proliferating DP-MSCs, BOECs and U373 at early passage were treated with either Doxo (model 2) or Aza (model 3) for 48 h before analysis.

### Characterisation of cultured cells

The DP-MSCs displayed spindle-shaped morphology under standard growing conditions, as expected of mesenchymal stem cells. Flow cytometric analysis of surface antigens revealed that over 95% of the cells positively expressed CD73, CD90, and CD105 antigens (Supplementary material Fig. S1–c). The cells did not express haematopoietic lineage markers: CD14, CD20, CD34 and CD45 (Supplementary material Fig. S1d). The results confirmed the DP-MSCs used in this study as mesenchymal stem cells.

BOECs were characterised for several endothelial and haematopoietic markers by flow cytometry. They expressed endothelial marker CD31 and VE-Cadherin (Supplementary material Fig. S1, Table C). As expected, no expression of the haematopoietic marker CD45 was found (Supplementary material Fig. S1, Table C). We also observed very low percentages of CD34^+^ and Tie-2^+^ in the BOECs. Besides, the cells displayed typical endothelial cobblestone-like morphology in culture and formed tube-like structures when differentiated on an extracellular matrix (left lower panel in supplementary material Fig. S1). Taken together, these results we confirmed that the BOECs are of endothelial lineage.

### Accumulation of senescent cells in long-term cultured MSCs

**Fig. 1:**
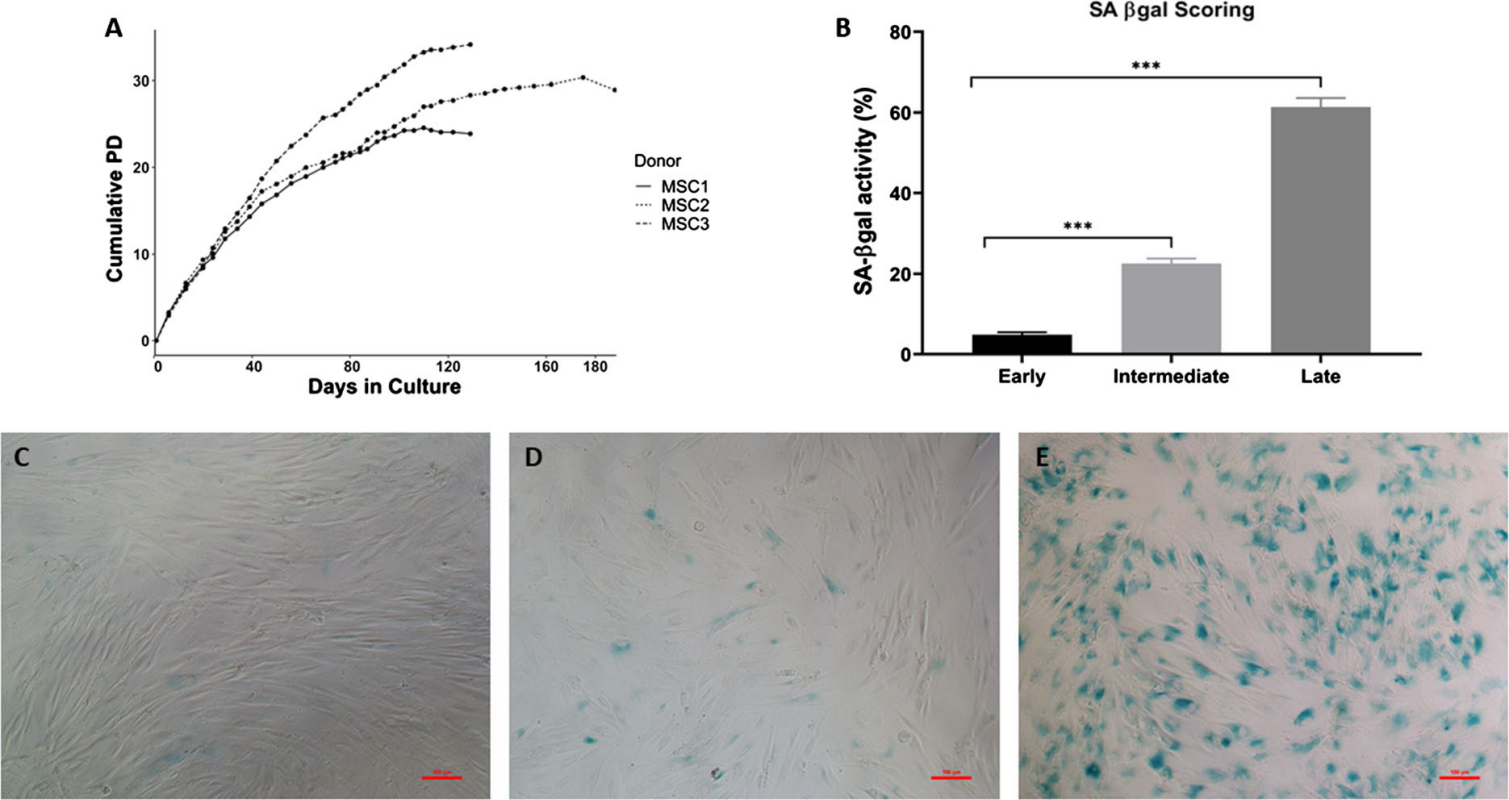
Long-term cultured MSCs under standard condition. **a** The growth kinetics for donors (n = 03) showing cumulative population doublings in culture. **b** The quantification of SA-β-Gal activity in cytochemical stained cells. The percentage of SA-β-Gal positive cells as an indication of SA-β-gal activity was quantified in n = 08 images per condition. Data presented as standard error mean bar and asterisks denote statistical significance (***p < 0.001) by ANOVA test. Representative images of cytochemical SA-β-Gal staining (**c**) early (**d**) intermediate (**e**) and late passages at scale bar of 100 µm

MSCs from three healthy individuals were maintained under standard medium until their proliferation rate significantly reduced. As expected, distinctive growth kinetics and progression toward the Hayflick limit for each sample were seen (Fig. 1a). We assessed the degree of senescence in this culture at three timepoints defined here as early, intermediate, and late passage based on their growth kinetics. Population doubling rate at these time points was significantly different between early and intermediate; early and late; and intermediate and late passage (Kruskal-Wallis test *p *< 0.001), confirming a decline in proliferation potential over time in culture.

The SA β galactosidase activity showed intense blue staining of lysosomal β galactosidase at later passages compared to the early stage (Fig. 1c). To quantify this activity, the number of SA-β-Gal positive (blue) cells versus the total number of cells in at least eight images per condition were counted manually. The percentage of SA-β-Gal positive cells indicates the level of SA-β-Gal activity in the cell population. The result confirms the accumulation of senescent cells with prolonged in vitro culture, peaking at late passage stage (Fig. 1b).

### Flow cytometric analysis of senescent cells

**Fig. 2:**
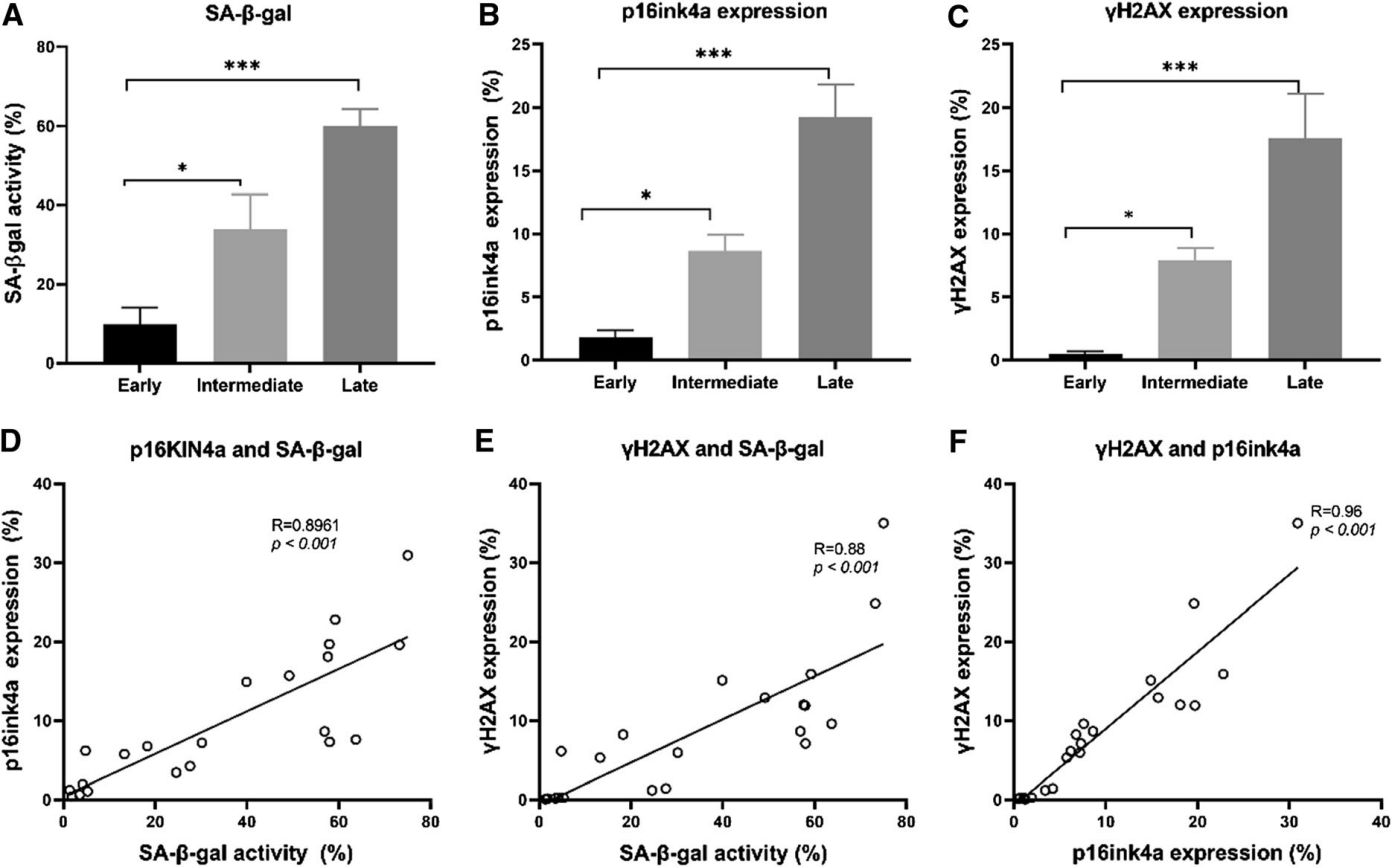
Detection and quantification of senescent cells by flow cytometry method in replicative senescent cells. Long term cultured MSCs (n = 07) were screened for level of senescence at early, intermediate, and late passage by flow cytometry-based method and displayed as the percentage of cells expressing the marker in each population. **a** SA-β-Gal activity level, **b** p16INKA4a expression and **c** DNA damage response marker (γH2AX) expression. The results are presented as the mean ± s.e.m. of three independent experiments and asterisks denote statistical significance (*p < 0.05; ***p < 0.001) by independent *t*-test The correlation of positive cells expressing senescence-associated markers **d** p16 and SA-β-Gal markers r = 0.896, *p *< 0.001, **e** γH2AX and SA-β-Gal markers r = 0.88, *p *< 0.001, **f** γH2AX and p16 markers r = 0.96, *p *< 0.001 by Spearman correlation

Next, we applied our flow cytometry multiparameter senescence assay to detect and quantify senescence in MSCs from seven different donors at early, intermediate, and late passages based on our previous grouping criteria from the growth kinetics. The SA-β-Gal activity was detected using the DDAOG substrate at 660 nm (APC channel) alongside with p16INK4a (p16) and γH2AX on PE and PECy7 channel respectively. Dead cells and debris were excluded from the analysis based on fixable viability dye FV450 staining and their light scatter feature. The gating strategy (Supplementary material Fig. S2) for identifying senescent cells was defined using the fluorescent minus one control for each marker (dye) as a benchmark. At least ten thousand viable cells were analysed per condition along with appropriate controls. Results obtained with cytochemical X-gal staining (Fig. 1b, c and e) were confirmed with the DDAOG staining by flow cytometry analysis (Fig. 2a). The percentage of cells expressing SA-β-Gal significantly increased over time in culture and the numbers in both assays were very similar. We also detected significantly elevated amounts of p16^INK4a^ and γH2AX, peaking in cells at the late passage (Fig. 2b and c). Notably, a strong positive correlation was found between cells expressing p16 and SA-β-Gal (R = 0.896, *p *< 0.001, Fig. 2d); γH2AX and SA-β-Gal (R = 0.88, *p *< 0.001, Fig. 2e); and γH2AX and p16 (R = 0.96, *p *< 0.001, Fig. 2f). This strong positive correlation suggests that an increase in one marker would generate a similar directional effect of a fixed proportion in the other marker.

From the flow data generated, additional senescence phenotypes can be readily assessed. For instance, we were able to estimate the cell size from the geometric mean of FSC vs. SSC. There is ample evidence that suggests a positive correlation between cell size and ageing in samples ranging from yeasts to mammalian cells (De Paiva et al. 2006). We observed via microscopy that the cell size at later passages was larger than at the earlier stage. This observation was confirmed by the cell size data extrapolated from our flow cytometer data (Supplementary material Fig. S3). Taken together, these observations confirmed the senescence level to be higher in the long-term cultured cells and hereafter refer to the late passage sample that has been in the replicative senescence stage.

Furthermore, we were able to discriminate the level of senescence in another cell type (BOECs) between early and late passage by using the same flow cytometry panel. Significantly higher SA-β-Gal expression was detected in cells at late passage compared to those at the early stage (Supplementary material Fig. S4a). We also observed significant differences in p16 and γH2AX expression between the early and late passaged BOECs (Supplementary material Fig. S4b, c). Interestingly, a similar strong positive correlation was seen between the markers (Supplementary material Fig. S4d–f) as observed in the MSCs. This result corroborates our findings with the stem cells and showed the markers as robust for senescence detection in BOECs as well as in MSCs.

### Induction of senescence by doxorubicin

**Fig. 3:**
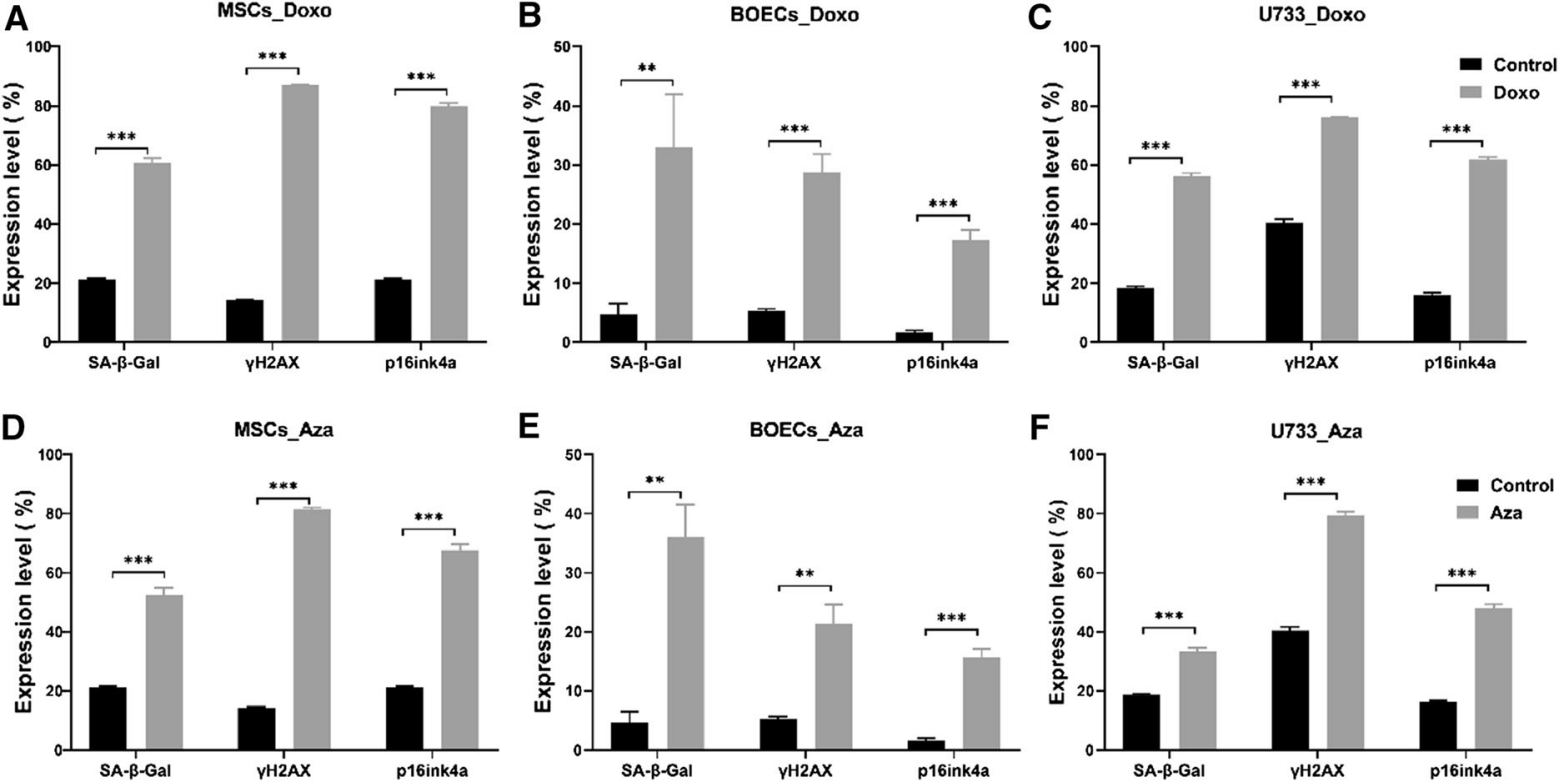
Quantification of senescence level in Doxo and Aza treated cells by flow cytometry. Quantification of senescence level in Doxorubicin and Aza- 2 deoxycytidines-treated cells by flow cytometry and displayed as the percentage of cells expressing the marker in each population. The upper panel shows the expression levels of SA-β-Gal, γH2AX, and p16^ink4a^ in MSCs (n = 03), BOECs (n = 04), and U373 (n = 03) cells treated with Doxo and their controls. The expression level of the three markers was significantly higher in the treated samples in comparison to non-treated samples. A similar result was seen in the Aza-treated sample (lower panel). The results are presented as the mean ± s.e.m. of three independent experiments. Asterisks denote statistical significance compared to early-stage (*p < 0.05; **p < 0.01; ***p < 0.001) from a *t-test* analysis

To further examine the application of this flow cytometry senescence detection panel, senescence was induced by Doxorubicin (Doxo) in three different cell types: mesenchymal stem cells, endothelial, and astrocyte cancer cell line. Actively dividing cells at early passages were treated with Doxo and cultured for an additional 5 days in fresh medium without Doxo. We observed a noticeable alteration in the cellular morphology of treated cells. The cells were enlarged and less defined in shape when compared to the non-treated cells (Supplementary material Fig. S5) on day 7, post-treatment. An increased level of SA-β-Gal activity, p16 expression and γH2AX expression were observed in Doxo-treated in comparison to non-treated cells. This result confirms the successful induction of senescence in MSCs (Fig. 3a), BOCEs (Fig. 3b), and U373 (Fig. 3c) cells by Doxo treatment.

### Induction of senescence by 5′-Aza-2′-deoxycytidine

**Fig. 4:**
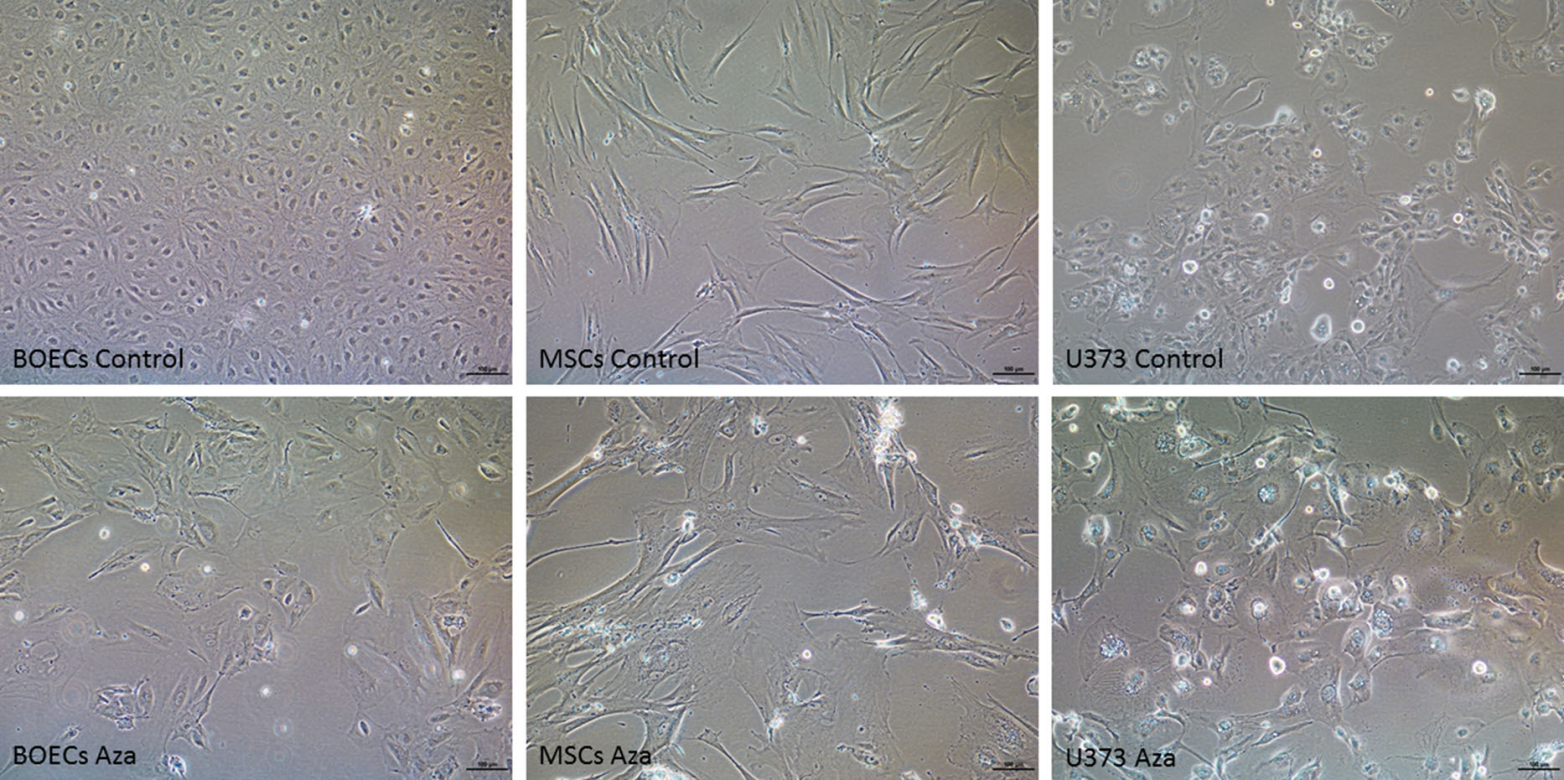
Morphological changes in 5′-Aza-2′-deoxycytidine treated cells. Cells were exposed to Aza for 48 h and imaged 5 days post-treatment. Scale bar = 100 µm

We proceeded to examine our last model for senescence induction and the consistency of the three biomarkers. Using a global DNA demethylase agent, senescence was induced in MSCs, BOECs, and U373. For this purpose, proliferating cells were treated with 5′-Aza-2′-deoxycytidine (5-AZA-dC), an FDA-approved anti-cancer drug (Ades and Metzger-Filho 2012) as described in the method section and screened for the presence of senescent cells post-treatment. To confirm whether Aza treatment could induce cellular senescence in MSCs, BOECs, and U373 cells, we investigated the phenotypic changes in these cells 5 days post-treatment. There was a noticeable morphological difference, the treated cells looked enlarged and flattened in shape (Fig. 4 -lower panel) when compared with the non-treated cells (Fig. 4 -upper panel). This observation mirrors morphological changes attributed to senescence, suggesting that the Aza treatment-induced senescence in these cells.

We then assessed SA-β-Gal activity using our flow cytometry-based approach as described in the previous section. A significantly higher SA-β*-*Gal activity was recorded in Aza treated samples than in the control for the three cell types (Fig. 3d–f). Also, the expression levels of p16 and γH2AX were measured in Aza-treated samples, which were significantly higher than in non-treated samples (Fig. 3d–f).

In addition to the three markers presented in this study, the endogenous autofluorescence which is known to accumulate with ageing (Bertolo et al. 2019) was quantified in the flow cytometric data. The autofluorescence signal in the 530/30 filter from treated (Doxo and Aza) MSCs was compared with that of the control. We observed a significantly increased level of autofluorescence in senescent cells in comparison with the control (Supplementary material Fig. S6). This finding is consistent with published studies where an elevated level of endogenous autofluorescence has been reported in senescent cells and tissues (Brunk and Terman 2002; Squirrell et al. 2012). In this study, we showed for the first time that Doxo and Aza induce senescence in BOECs. Notably, BOECs displayed similar senescence phenotypes as seen in MSCs both in the long term cultured and, in the senescence induced cells. We observed the up-regulation of SA-β-Gal, p16 expression, and significantly higher accumulation of DNA damage response in treated samples than in the controls.

### Validation of flow cytometric senescence detection approach

**Fig. 5:**
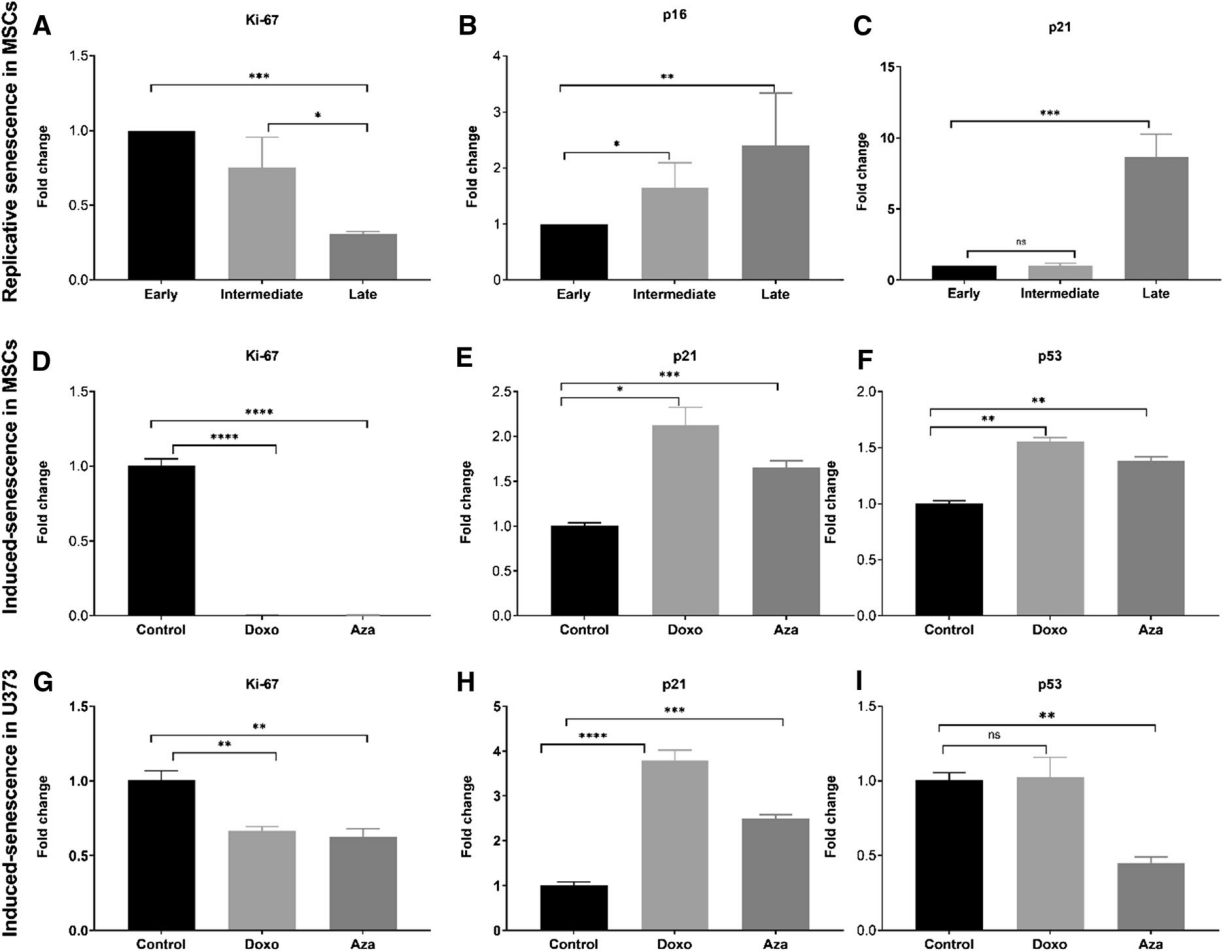
Quantification of senescence markers by RT-qPCR. Real-time qPCR was used to measure the expression levels of Ki-67 (**a**), p16 (**b**), and p21 (**c**) in replicative senescence. For the induced senescence in MSCs, the expression levels of Ki-67 (**d**), p16 (**e**) and p53 (**f**) were quantified. Similarly, the expression levels of Ki-67 (**g**), p16 (**h**) and p53 (**i**) were measured in U373. The results are presented as the mean ± s.e.m. of three independent experiments. Asterisks denote statistical significance compared to control—early stage for replicative senescence, and non-treated (control) for premature senescence (*p < 0.05; **p < 0.01; ***p < 0.001, ns = not significant) from a *t-test* analysis

Finally, two independent methods were used to confirm the detection of senescence by the flow cytometric assay on the replicative and induced senescent cell samples in addition to previously described cytochemical SA-β-Gal staining. The rationale was to profile two main components associated with senescence: cell cycle arrest and the secretome to confirm induction of senescence in the cells and changes in the level of senescent cells. The first approach was based on the highly sensitive and precise quantitative real-time PCR (qPCR) to measure the expression of four well-established senescence biomarkers: p16, p21, Ki-67, and p53. The expression level of the proliferation marker Ki-67 was down-regulated in the replicative (Fig. 5a) and in the induced senescence (Fig. 5d, g), suggesting cell cycle arrest in these cells. In the replicative senescence, our result showed a gradual but steady increase in p16 expression over time in culture (Fig. 5b) and in BOECs (Supplementary material Fig. 7s). We likewise observed an increase in the level of p21 expression in all three senescence models studied (Fig. 5c, e and h). Similarly, the expression level of p53 was up-regulated in the Doxo-induced senescence, but no significant difference was seen between the control and Doxo-treated in the U373 cell line (Fig. 5i), which may suggest p53-independent senescence induction by Doxo in U373. Taken together, these results confirm the induction of senescence in the cells and show concordance with the results from the flow cytometric approach.

**Fig. 6:**
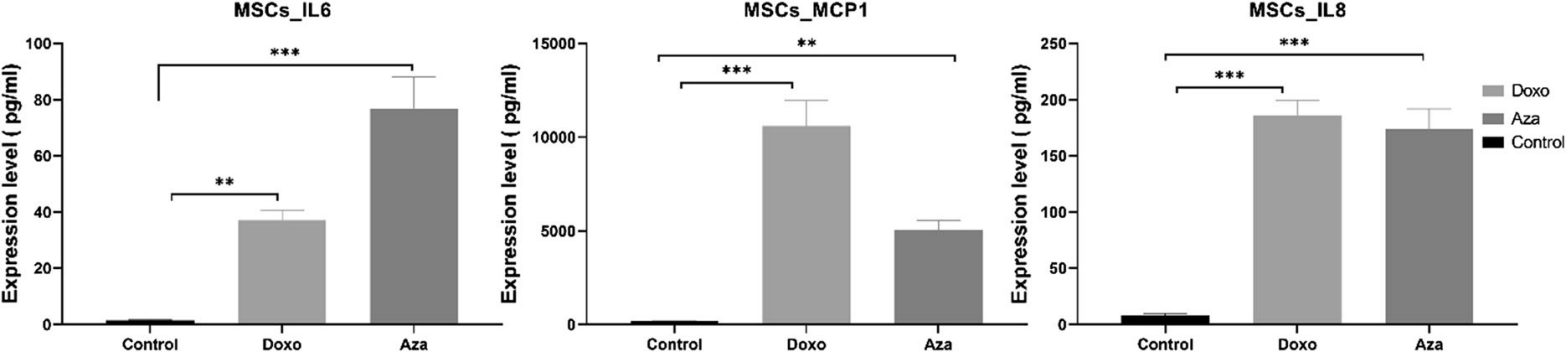
Expression of the SASP cytokines IL6, MCP1 and IL8 in Doxo and Aza–induced senescence. The expression level of IL6, MCP1, and IL8 was significantly increased in both Aza and Doxo treated cells compared to the control (non-treated). The results are presented as the mean ± s.e.m. Asterisks denote statistical significance (*p < 0.05; **p < 0.01; ***p < 0.001) from ANOVA test n = 03 per condition and 3 replicates

The second approach was to measure the expression levels of the senescence-associated secretory phenotype (SASP) IL6, MCP1 and IL8 in the supernatant of both the Doxo and Aza-induced-senescent MSCs. Results from this experiment showed a significant increase in the expression of IL6, MCP1 and IL8 secreted by Doxo and Aza-treated cells in comparison to the control (Fig. 6).

## Discussion

Despite great advancement in our understanding of senescence, a reliable universal biomarker capable of accurately discriminating senescent cells has remained elusive. Thus, a deeper understanding of the biological implications of the senescence phenotype and its therapeutic potentials and risk has been hindered. In this paper, we described a three-colour flow cytometric panel that simultaneously quantifies multiple senescence markers at high throughput. This panel was informed by senescence biomarkers with consistent results from the literature, and a dye that supports detection of SA-β-Gal in living cells but does not interfere with the increasing autofluorescence of senescent cells.

There is now substantial evidence that most of the beneficial effects of MSCs in regenerative medicine stem from the broad repertoire of secreted trophic factors that modulate various functions, such as anti-inflammatory and immunomodulation processes. It is also known that senescent cells secrete a myriad of factors such as growth factors and pro-inflammatory cytokines with potent autocrine and paracrine activities (Coppé et al. 2010). We report significant up-regulation of IL8, IL6, and monocyte chemotactic protein-1 (MCP1) following senescence induction in MSCs. Notably, these cytokines can exacerbate the inflammation response often associated with age-related diseases (López-Otín et al. 2013). Senescence activation pathways and phenotypes are well documented in the literature as independent of the tissue source and the stressor (Campisi 2013; Fagagna et al. 2003; Fumagalli et al. 2014; Stein et al. 1999). Hence, the similarity in the phenotypes and expression pattern of SA-β-gal, p16 and γH2AX in senescent BOECs with those of MSCs agreed with what is known in the literature. This study adds to our sparse knowledge of BOECs’ senescence and provides a tool to track senescence in BOECs.

In an era of rapidly expanding regenerative medicine applications, mesenchymal stem cells and endothelial cells play an important role in revolutionising these therapies for diseases. High demand, enthusiasm for regenerative medicine, and regulatory approvals (Shapiro et al. 2019) are positive signals of future increase in the proliferation of cell-based therapies. Recent studies have highlighted the risk associated with senescent cells in cell therapy. For instance, Xu et al. reported osteoarthritis-like condition in the legs of mice transplanted with senescent cells (Xu et al. 2017). In another study, it was shown that transplanting relatively small numbers of senescent cells into the abdomens of young mice was enough to induce systemic disability and disease (Xu et al. 2018). Considering the potential deleterious risks that senescent cells may pose, it is increasingly important to monitor the senescence phenotypes in therapeutic cell preparations. A growing inclination in the field is to include cellular senescence assessment as part of quality control for reliable and reproducible cellular therapy (Turinetto et al. 2016).

Different methods have been proposed and tested to track senescence in the cell population with the goal of detection and quantification (Turinetto et al. 2016). The cumulative population doubling method, for instance, is simple to implement, but its sensitivity in predicting cells approaching a senescent state is limited by factors such as large donor variations, donor age, and cells lost via apoptosis during passaging (Cristofalo et al. 1998). On the other hand, the popular cytochemical staining of SA-β-gal is more robust in detecting enlarged cells (which are usually late senescent cells) but precludes the detection of early senescent cells (Turinetto et al. 2016). Recently, the flow cytometric-based approach is emerging as a fast and reliable method to quantify senescent cells. For instance, in 2017, Althubiti and Macip proposed the use of the extracellular markers to characterise senescent cells (Althubiti and Macip 2017). There have also been fluorescent-based methods for detecting SA-β-Gal, with the advantage of incorporation of other senescent markers and improved sensitivity (Bertolo et al. 2019; Flor et al. 2016; Höhn and Grune 2013). In this study, we proposed a tool to monitor senescence phenotypes in expanding *ex vivo* cells and in cancer cells by combining the advantages of flow cytometry with the high sensitivity of fluorescence detection.

For SA-β-gal activity detection by flow cytometer, a cell-permeable β*-*galactosidase DDAOG substrate was chosen instead of the commonly used C_12_-FDG. DDAOG hydrolyses to DDAO upon cleavage by β-galactosidase, resulting in a far-red fluorescence detectable by most flow cytometers. The choice of DDAOG prevents cellular autofluorescence (AF) cross-talk, a major drawback with using C_12_-FDG or other fluorogenic substrates such as the SPiDER-βGal in SA-β-Gal detection, which tends to emit at around a 550 nm wavelength (Flor et al. 2016; Höhn and Grune 2013). DDAOG is also a preferred option for detecting SA-β-Gal activity in vivo, as the shift to the far-red fluorescence allows a clear detection against the probe background (Tung et al. 2004). Our approach incorporates the sensitivity and reliability of the DDAOG substrate in senescence detection with other senescent cell biomarkers. Furthermore, the design of the panel allows the signal from the age-related pigment lipofuscin to be captured in a separate channel as an additional data point for senescence detection (Bertolo et al. 2019).

Replicative senescence of mesenchymal stem and endothelial cells was generated from a very early passage by a prolonged culture under standard growing conditions. The developed assay was sensitive enough to discriminate the level of senescence at different passages of culture. Previous studies have demonstrated that senescent cells accumulate in ageing tissues and long-term cultured cells (Angello et al. 1987). This is consistent with our finding that senescent cells accumulate over time in culture. This opens the possibility of using this assay to quantify senescent cell accumulation in cells expanded for therapeutic purposes.

References from the literature indicate that most senescent cells express p16^INK4a^, which is mostly absent in quiescent and terminally differentiated cells, making it a valuable senescence biomarker (Stein et al. 1999; Zindy et al. 1997). Likewise, senescent cells are known to harbour irreparable DNA damage in their nucleus (Turinetto and Giachino 2015). This finding in this study agrees with previous studies where the accumulation of DNA damage in senescent cells in tissues and organs of older organisms increases (Fumagalli et al. 2014; López-Otín et al. 2013). We demonstrated a strong positive correlation between these markers and SA-β-Gal at a single-cell resolution in both replicative and stress-induced senescence. SA-β-gal activity strongly correlates with the accumulation of DNA damage and p16 expression in both cell types.

The multiparameter flow cytometry assay for senescence detection described in this study can be used to track senescence in living cells based on the DDAOG substrate. It also allows the generation of additional data in a non-intrusive manner. For example, the accumulation of lipofuscin can be estimated in the green channel and the estimation of cell size possible from the flow cytometric data.

Senescence has gained increasing interest from the cancer therapeutic perspective, as a promising strategy to inhibit the proliferation of cancer cells. Therapy-induced senescence (TIS) has been reported in cancer cell lines treated with anticancer drugs, as well as in tumour samples from patients exposed to chemotherapy (Braig et al. 2005). A recent study showed an improved outcome when TIS lymphoma cells were eliminated in mice (Dörr et al. 2013). Hence, tools that would allow the identification and quantification of TIS post-treatment are needed. In this study, we showed that the flow cytometric-based senescence detection assay can be used to detect and quantify senescent cells in the U373 human glioblastoma cell line following treatment with anticancer drugs Doxo and Aza. With this tool, it is possible to monitor the senescence level in patients’ cells, such as endothelial cells, following anti-cancer drug treatment.

Our results show that the flow-cytometric method can be used to discriminate a wide variety of senescent cells in actively dividing cell populations as effectively as conventional senescence detection methods. Considering the sensitivity, lower labour, and high throughput platform enabled by flow cytometry, we strongly recommend the flow cytometric- based method for quantifying senescent cells in culture and in *ex vivo* cells. This method can also be used to enhance high throughput screenings, relevant to senolytic and anti-ageing drug development. Another possible application of this method could entail screening senescent cells from cell-based therapeutic products.

## Electronic supplementary material

Below is the link to the electronic supplementary material.

Electronic supplementary material 1 (PPTX 8065 kb)
