# Supplementary material for: Multiparameter flow cytometric detection and quantification of senescent cells in vitro
Source: Biogerontology. 2020 Aug 10;21(6):773–86. doi: 10.1007/s10522-020-09893-9 (PMC7541365; doi:10.1007/s10522-020-09893-9)
Supplement: Supplementary file 1 — Electronic supplementary material 1 (PPTX 8065 kb) [file 10522_2020_9893_MOESM1_ESM.pptx]

## Slide 1
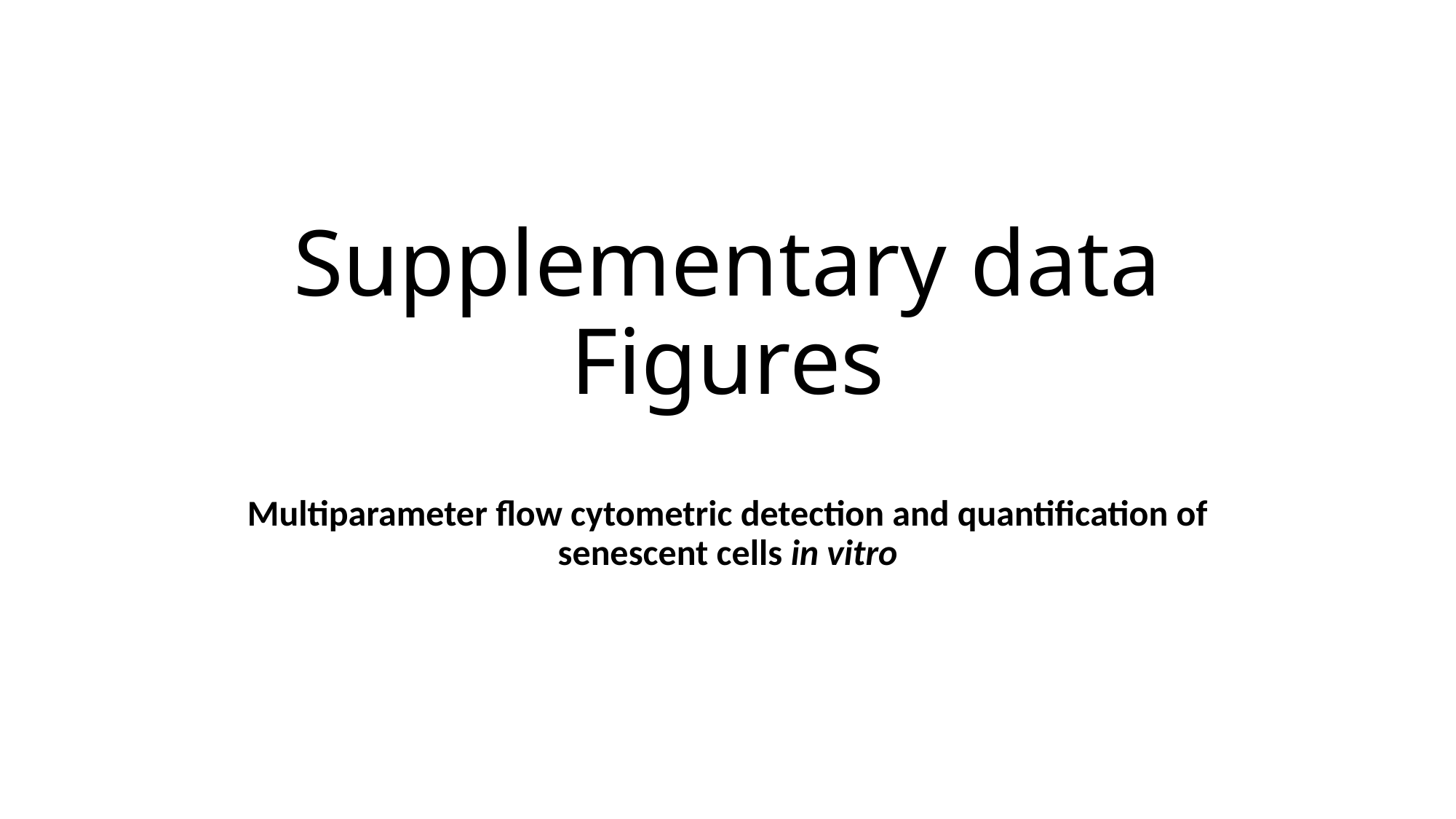

# Supplementary data Figures
Multiparameter flow cytometric detection and quantification of senescent cells in vitro

## Slide 2
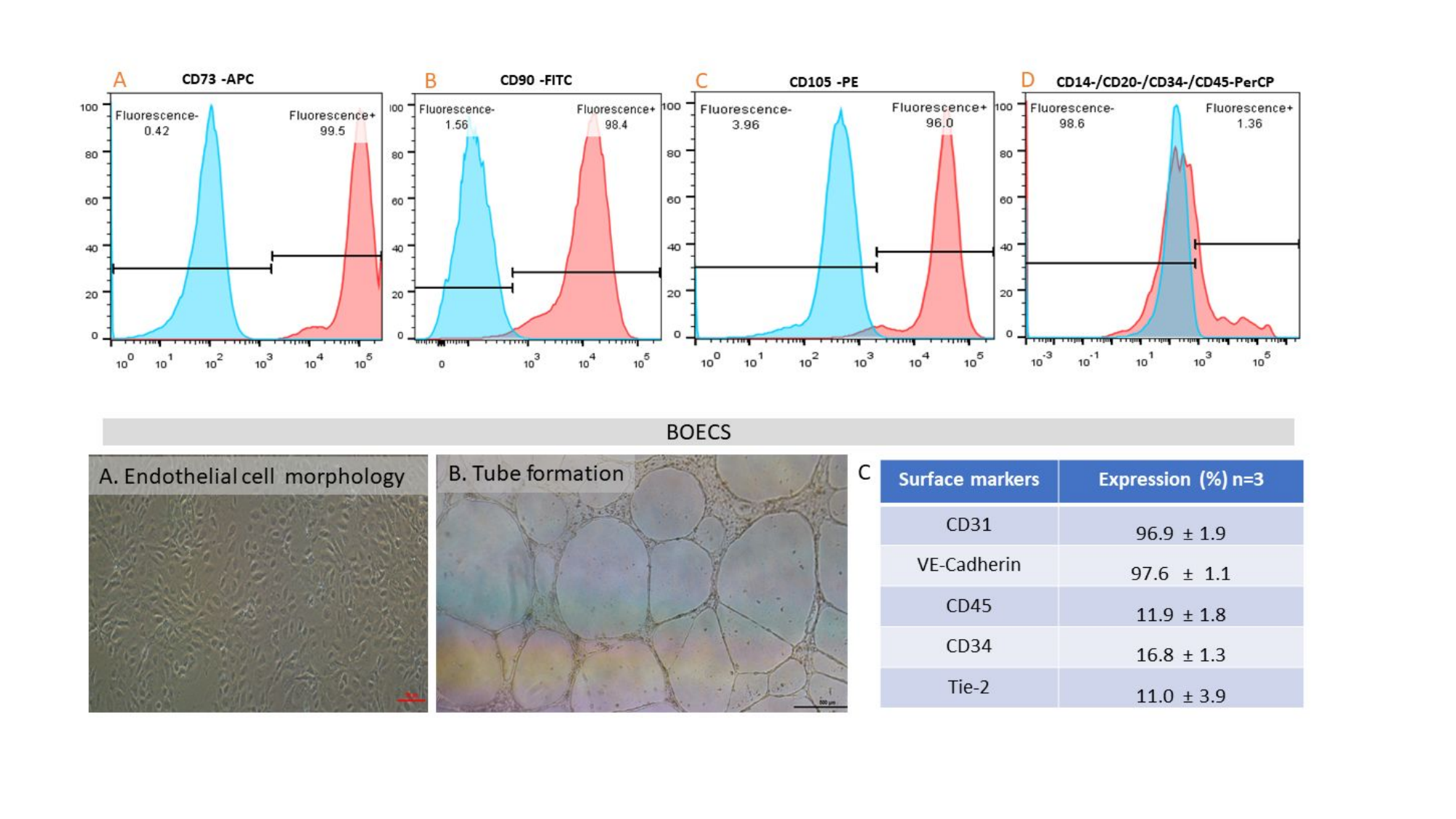

## Slide 3
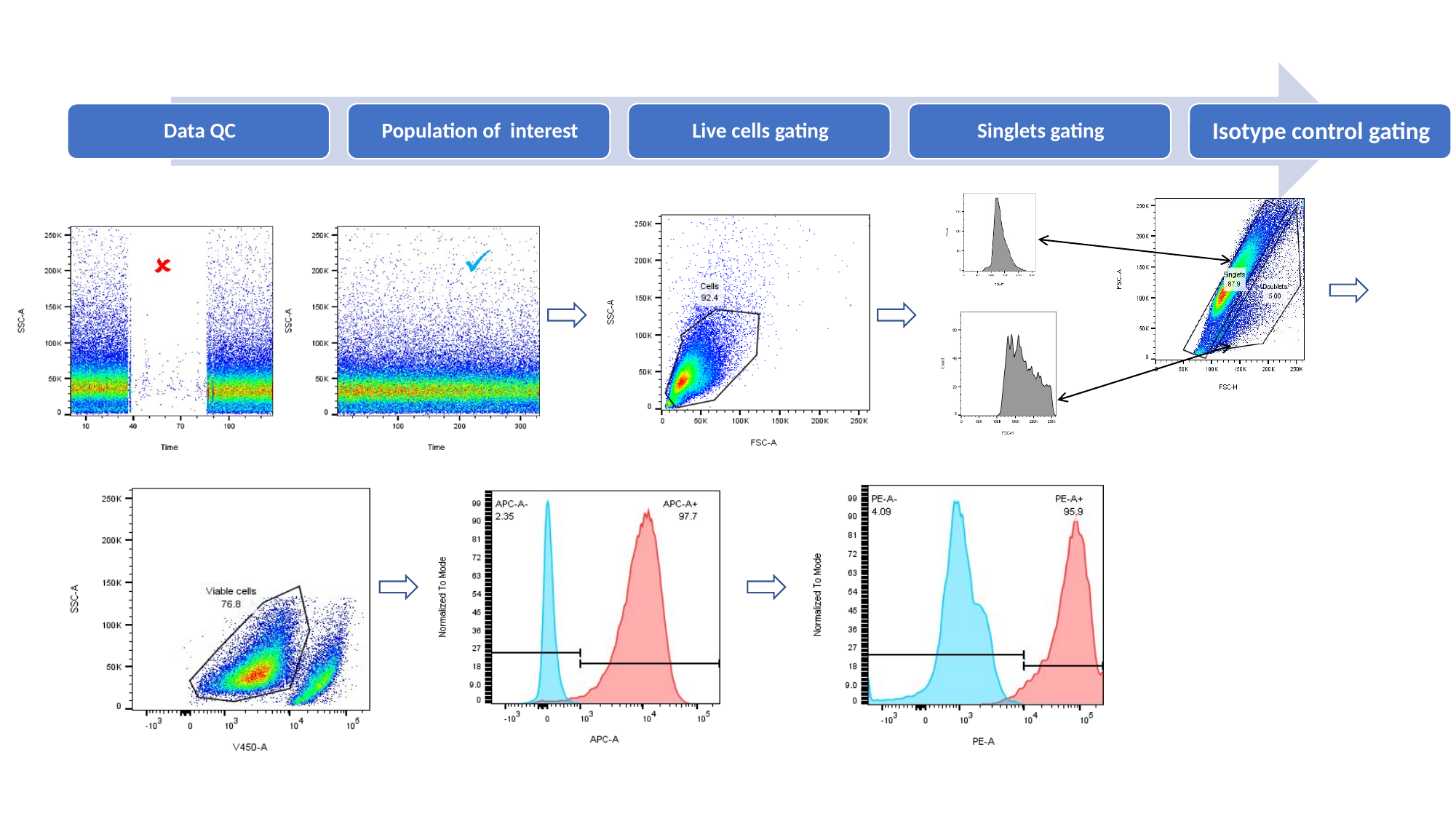

## Slide 4
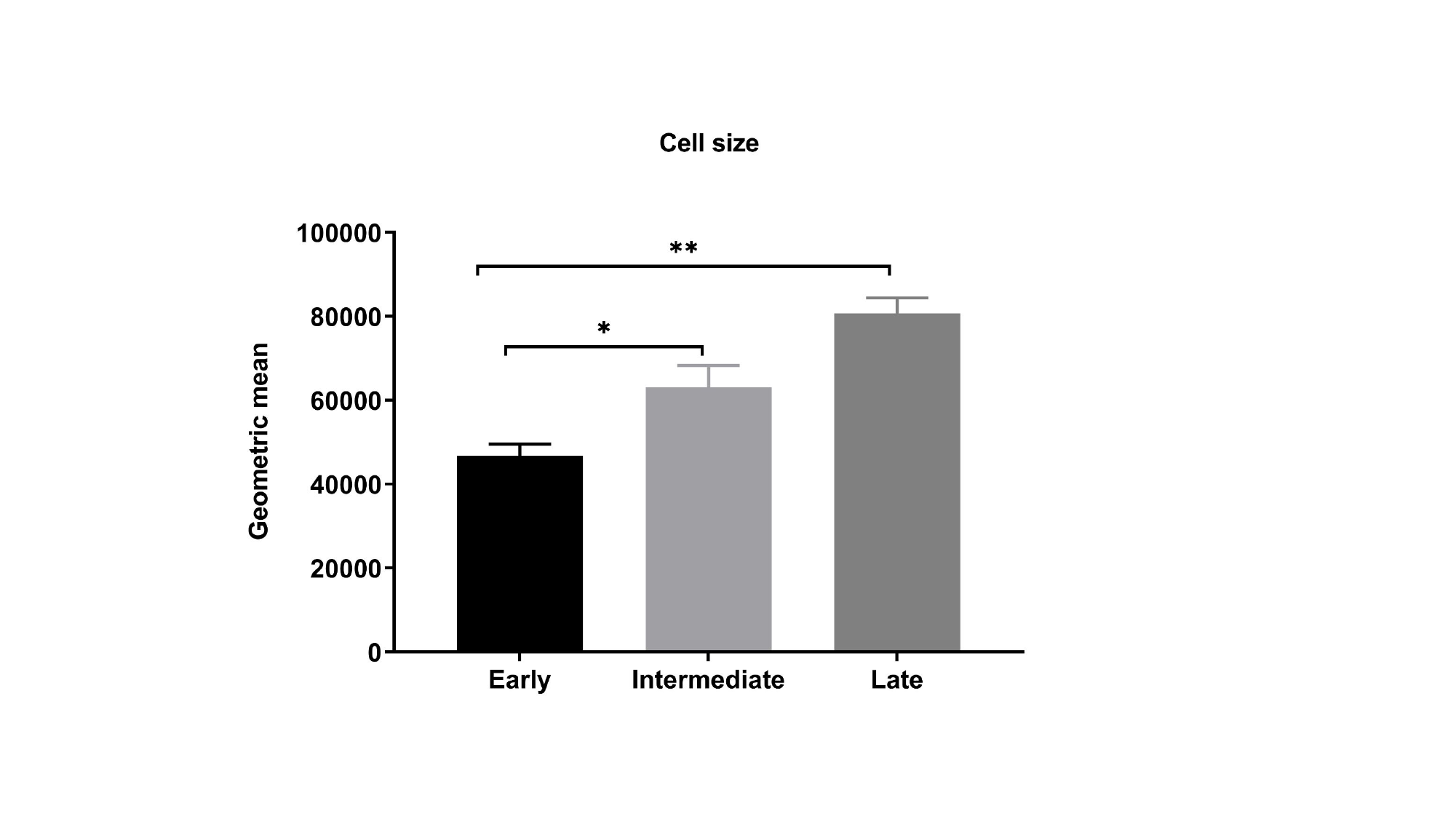

## Slide 5
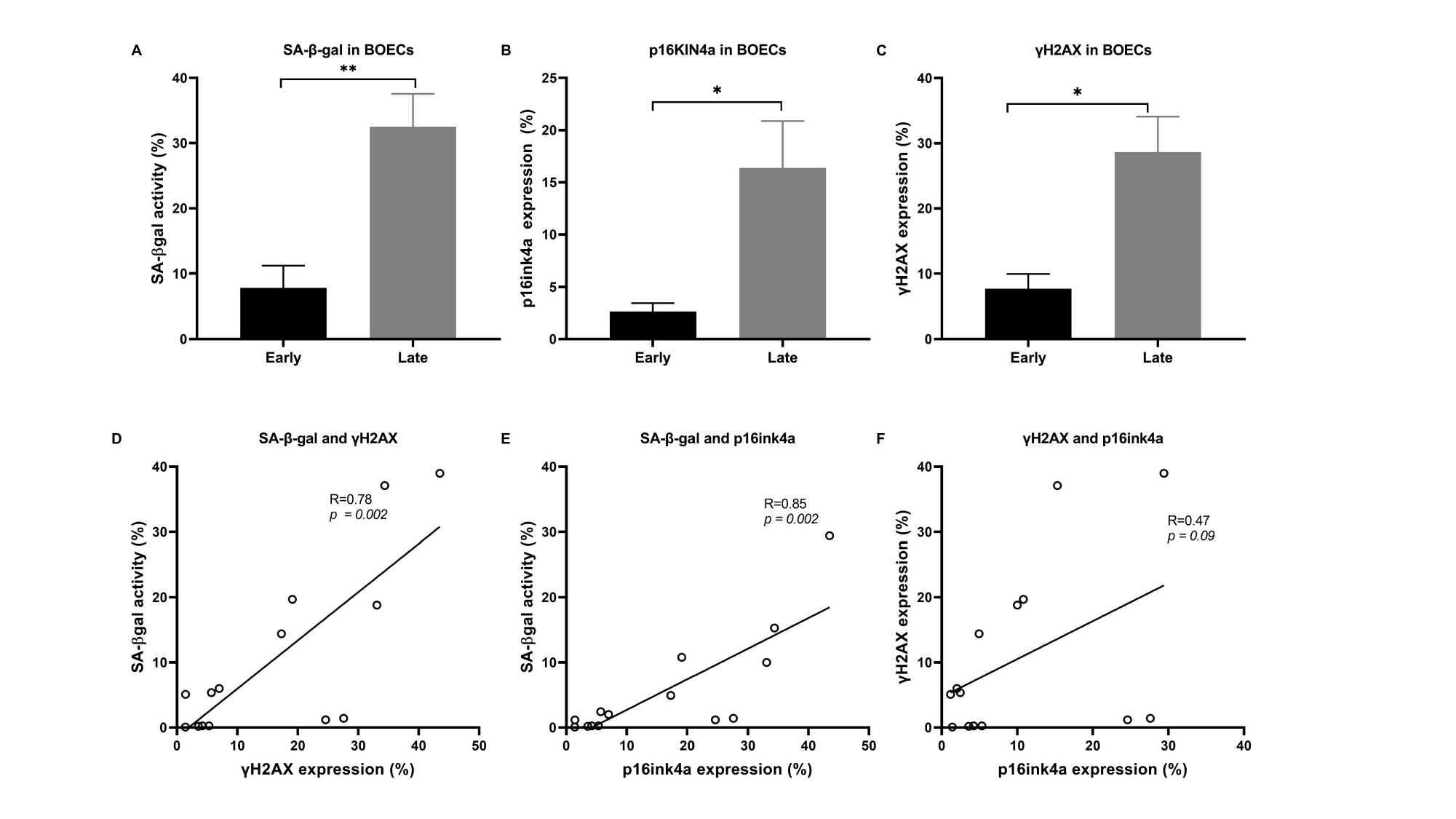

## Slide 6
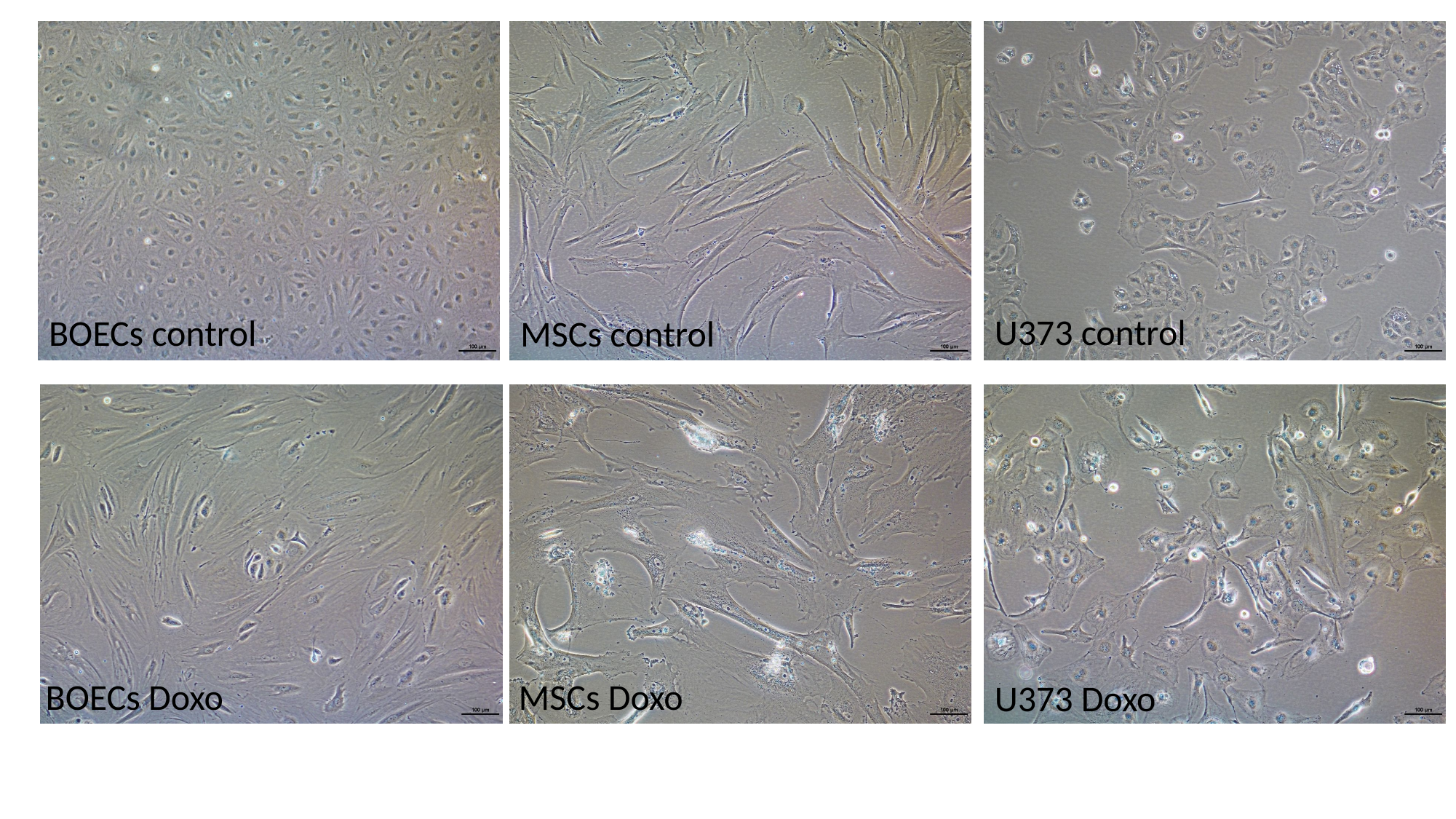

U373 control
BOECs control
MSCs control
MSCs Doxo
BOECs Doxo
U373 Doxo

## Slide 7
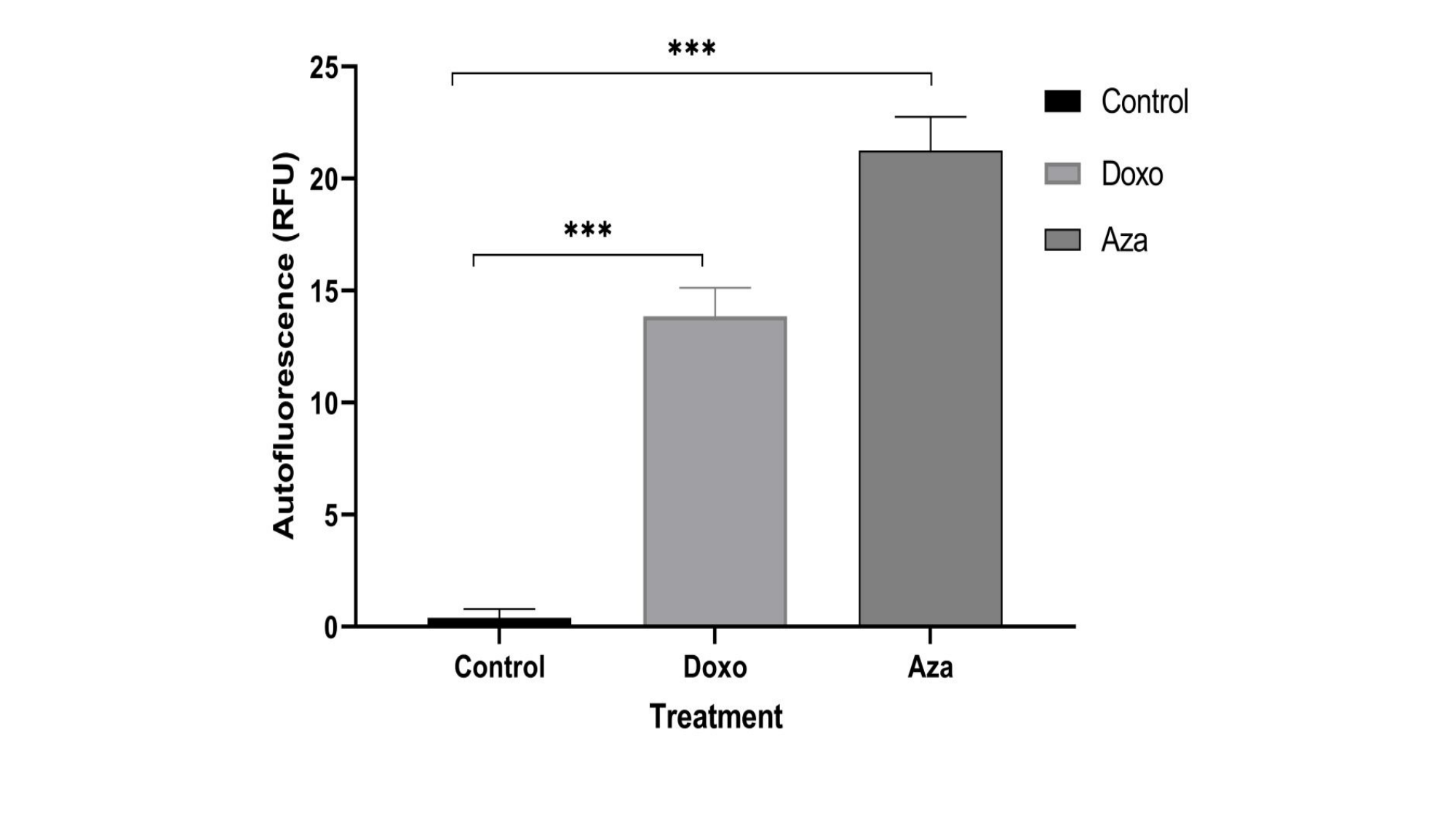

## Slide 8
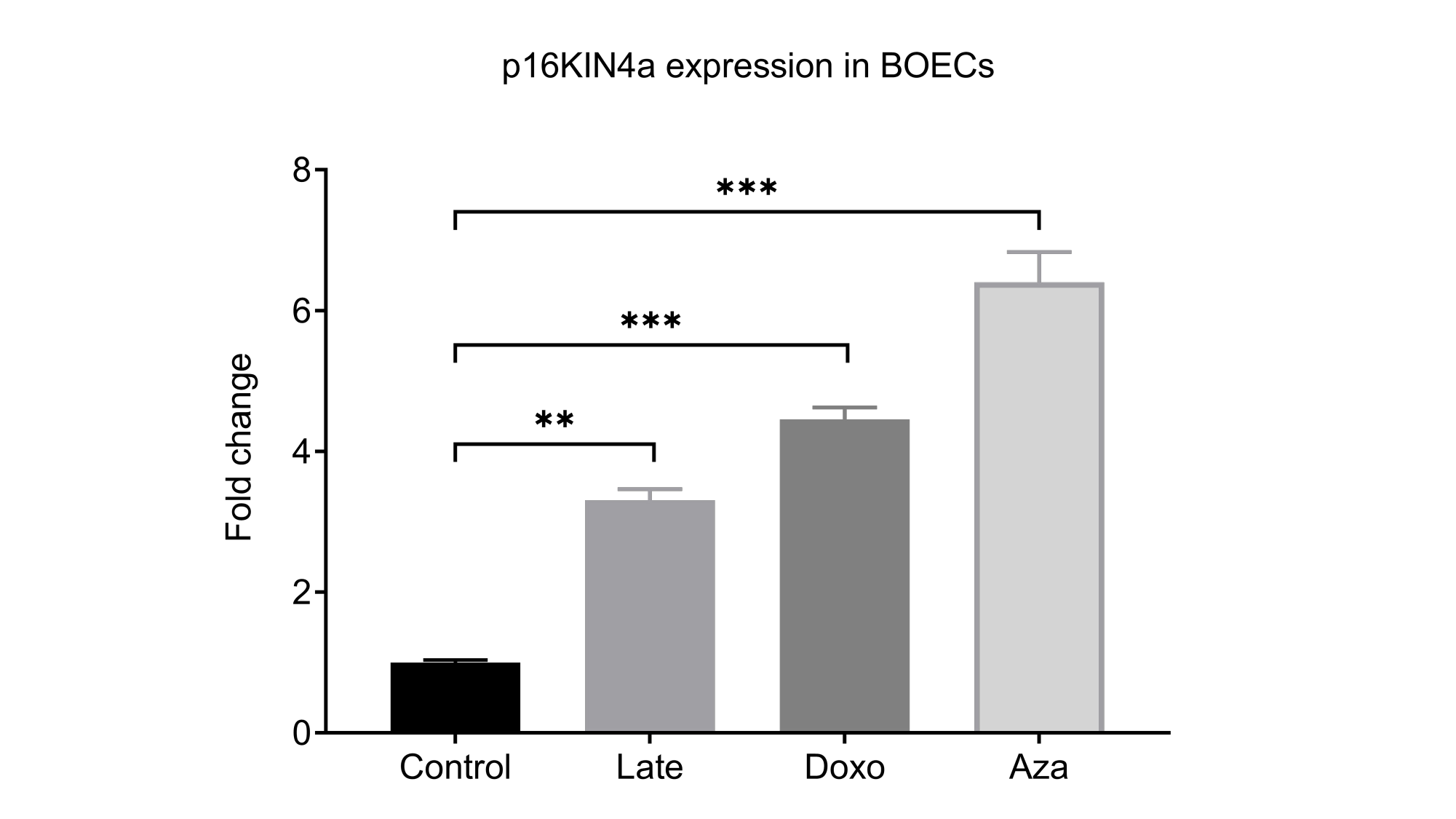

## Slide 9
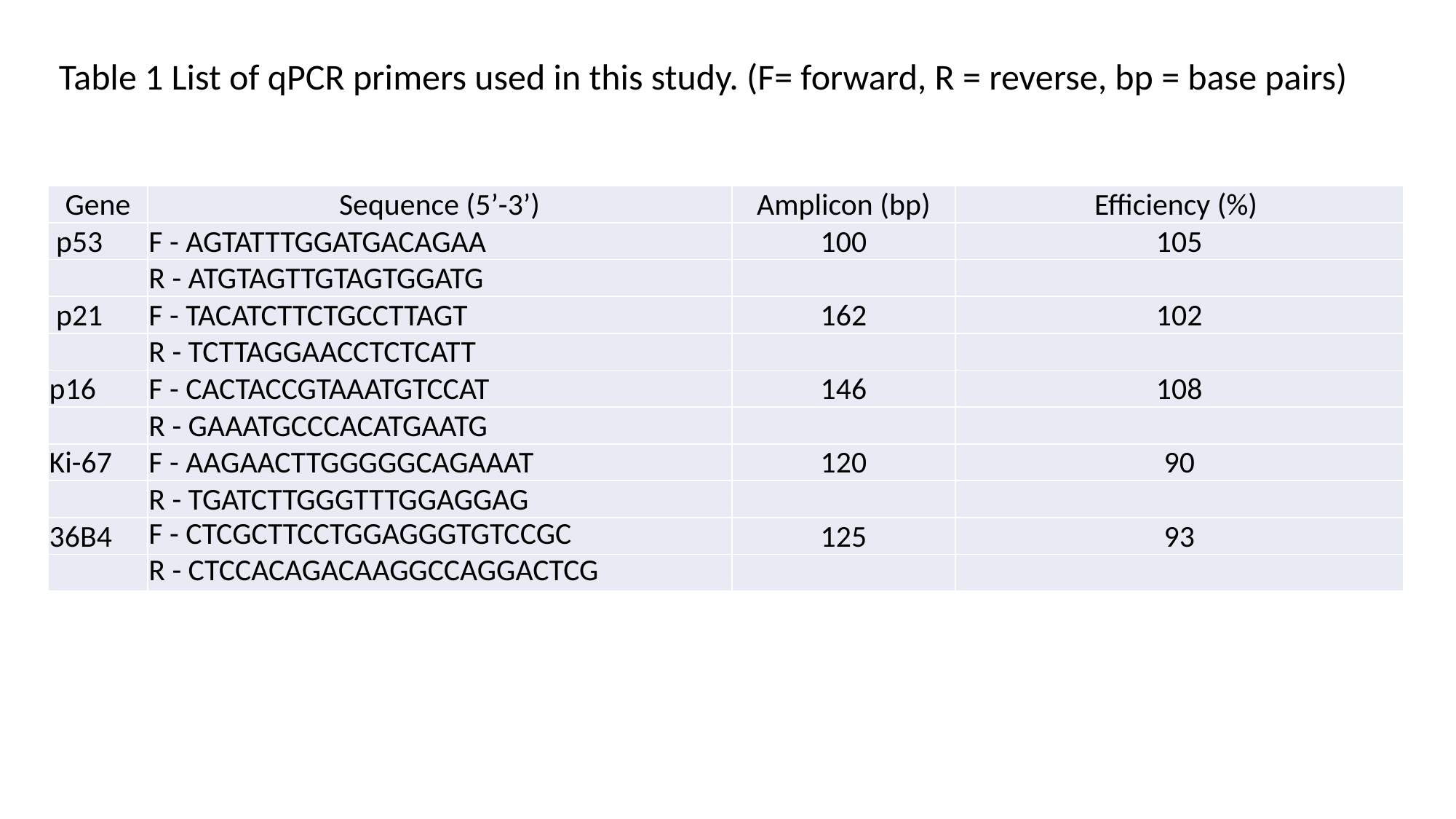

Table 1 List of qPCR primers used in this study. (F= forward, R = reverse, bp = base pairs)
| Gene | Sequence (5’-3’) | Amplicon (bp) | Efficiency (%) |
| --- | --- | --- | --- |
| p53 | F - AGTATTTGGATGACAGAA | 100 | 105 |
| | R - ATGTAGTTGTAGTGGATG | | |
| p21 | F - TACATCTTCTGCCTTAGT | 162 | 102 |
| | R - TCTTAGGAACCTCTCATT | | |
| p16 | F - CACTACCGTAAATGTCCAT | 146 | 108 |
| | R - GAAATGCCCACATGAATG | | |
| Ki-67 | F - AAGAACTTGGGGGCAGAAAT | 120 | 90 |
| | R - TGATCTTGGGTTTGGAGGAG | | |
| 36B4 | F - CTCGCTTCCTGGAGGGTGTCCGC | 125 | 93 |
| | R - CTCCACAGACAAGGCCAGGACTCG | | |
